# Nailfold capillaroscopy and deep learning in diabetes

**DOI:** 10.1111/1753-0407.13354

**Published:** 2023-01-15

**Authors:** Reema Shah, Jeremy Petch, Walter Nelson, Karsten Roth, Michael D. Noseworthy, Marzyeh Ghassemi, Hertzel C. Gerstein

**Affiliations:** ^1^ Population Health Research Institute, McMaster University and Hamilton Health Sciences Hamilton Ontario Canada; ^2^ Centre for Data Science and Digital Health Hamilton Health Sciences Hamilton Ontario Canada; ^3^ Institute for Health Policy, Management and Evaluation University of Toronto Toronto Ontario Canada; ^4^ Division of Cardiology McMaster University Hamilton Ontario Canada; ^5^ Department of Statistical Sciences University of Toronto Toronto Ontario Canada; ^6^ Cluster of Excellence Machine Learning University of Tübingen Tübingen Germany; ^7^ Electrical and Computer Engineering McMaster University Hamilton Ontario Canada; ^8^ McMaster School of Biomedical Engineering Hamilton Ontario Canada; ^9^ Department of Radiology McMaster University Hamilton Ontario Canada; ^10^ Vector Institute Toronto Ontario Canada

**Keywords:** diabetes diagnosis, machine learning, nailfold capillary, retinopathy, 糖尿病诊断, 视网膜病变, 甲襞毛细血管, 机器学习

## Abstract

**Objective:**

To determine whether nailfold capillary images, acquired using video capillaroscopy, can provide diagnostic information about diabetes and its complications.

**Research Design and Methods:**

Nailfold video capillaroscopy was performed in 120 adult patients with and without type 1 or type 2 diabetes, and with and without cardiovascular disease. Nailfold images were analyzed using convolutional neural networks, a deep learning technique. Cross‐validation was used to develop and test the ability of models to predict five5 prespecified states (diabetes, high glycosylated hemoglobin, cardiovascular event, retinopathy, albuminuria, and hypertension). The performance of each model for a particular state was assessed by estimating areas under the receiver operating characteristics curves (AUROC) and precision recall curves (AUPR).

**Results:**

A total of 5236 nailfold images were acquired from 120 participants (mean 44 images per participant) and were all available for analysis. Models were able to accurately identify the presence of diabetes, with AUROC 0.84 (95% confidence interval [CI] 0.76, 0.91) and AUPR 0.84 (95% CI 0.78, 0.93), respectively. Models were also able to predict a history of cardiovascular events in patients with diabetes, with AUROC 0.65 (95% CI 0.51, 0.78) and AUPR 0.72 (95% CI 0.62, 0.88) respectively.

**Conclusions:**

This proof‐of‐concept study demonstrates the potential of machine learning for identifying people with microvascular capillary changes from diabetes based on nailfold images, and for possibly identifying those most likely to have diabetes‐related complications.

## Graphical abstract

**Highlights**

- Nailfold capillary images were obtained by performing nailfold video capillaroscopy in 120 adult patients with and without diabetes.
- Over 5000 photographs were analyzed using machine‐learning approaches, and these models were clearly able to distinguish people based on diabetes status.
- This proof‐of‐concept study demonstrates the potential of machine learning for identifying people with microvascular capillary changes from diabetes based on nailfold images and for possibly identifying those likely to have diabetes‐related complications.

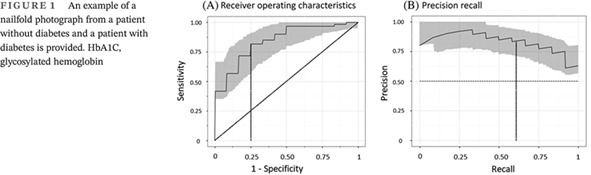

## 摘要

**目的:**探讨视频毛细血管镜获取的甲襞毛细血管图像能否提供糖尿病及其并发症的诊断信息。

**方法:**对120例成人患者进行视频甲襞毛细血管镜检查, 患者有或无1型或2型糖尿病, 有或无心血管疾病。使用卷积神经网络(CNNs), 一种深度学习技术对甲襞图像进行分析。交叉验证用于开发和检验模型预测5种预设状态(糖尿病、高HbA_1c_、心血管事件、视网膜病变、白蛋白尿和高血压)的能力。通过评估受试者工作特征曲线下面积(AUROC)和精确召回曲线(AUPR)来评估每个模型对特定状态的性能。

**结果:**120名参与者共获得5,236张甲襞图像(平均每位参与者44张), 所有图像均可用于分析。模型能够准确识别糖尿病的存在, AUROC为0.84 (95% CI 0.76, 0.91), AUPR为0.84 (95% CI 0.78, 0.93)。模型还能够预测糖尿病患者的心血管事件史, AUROC为0.65 (95% CI 0.51, 0.78), AUPR为0.72 (95% CI 0.62, 0.88)。

**结论:**这项概念验证研究证明了机器学习在基于甲襞图像从糖尿病中识别微血管毛细血管改变人群的潜力, 并可能识别出最有可能发生糖尿病相关并发症的人群。

## 1 INTRODUCTION

People with diabetes are at high risk for a wide variety of serious health consequences involving multiple organ systems throughout the body.^1^, ^2^, ^3^ The risk of these consequences generally rises with the degree of hyperglycemia, duration of diabetes, age, and the presence of other risk factors such as hypertension and smoking.^1^, ^4^ Although the underlying reasons for these diabetes‐related health consequences are not clearly elucidated, there is growing recognition that many are linked to structural and functional abnormalities in the microcirculation of the relevant organ.^5^, ^6^, ^7^ The most pathognomonic of these is the retina, likely because its vascular bed can be visualized by ophthalmoscopy. The relationship between morphological changes in arteriolar and capillary beds in the retina and the future occurrence of most of the long‐term consequences of diabetes has focused attention on retinal imaging as a way of discriminating between high‐ and low‐risk patients.^5^ Moreover, the availability of large repositories of retinal images linked to phenotypic information has facilitated the application of image processing and machine‐learning technology to optimize the diagnostic and prognostic value of retinal images.^8^

Optimal visualization of the retinal capillaries requires the topical application of mydriatic agents to the eyes, expensive cameras, and either the injection of dye or the use of optical coherence tomography. These requirements have restricted the use of retinal photography to specialized clinical settings. However, similar information may reside in other vascular beds such as the one at the nailfolds, which can be easily and noninvasively photographed using nailfold video capillaroscopy. The relative ease of collecting and digitally storing such images from more than one finger presents an opportunity to determine whether the morphology of the capillaries located there contain diagnostic and/or prognostic information relevant to diabetes.

This possibility was assessed in a proof‐of‐concept study in which nailfold video capillaroscopic images were obtained from 120 patients with and without diabetes and then analyzed with a common machine‐learning technique.

## 2 MATERIALS AND METHODS

### 2.1 Study design, setting, and population

This was an observational cross‐sectional study of 120 participants with and without diabetes and with and without cardiovascular disease. Participants aged 35–75 years were recruited through the Boris Clinic at McMaster University and the Heart Investigation Unit at Hamilton Health Sciences in Hamilton, Ontario, Canada. Recruitment was stratified by diabetes and cardiovascular disease status, such that there were equal numbers of patients with and without diabetes and with and without cardiovascular disease. Those with a history of connective tissue disease and Raynaud's syndrome were excluded from our study, as were people with bilateral finger or hand deformity, which restricted the ability to photograph the nail beds. Patients with diabetes were required to have a glycosylated hemoglobin (HbA1c) ≥6.5% and duration of diabetes of at least 10 years. The study was approved by the Hamilton Integrated Research Ethics Board and all participants provided written informed consent.

### 2.2 Imaging of nailfold capillaries

Nailfold capillary images were taken using the Optilia Video Capillaroscope (Optilia Instruments AB, Sweden) at a magnification of 200x and stored and analyzed using the Optipix software.^9^ Patients were first acclimatized for a minimum of 15 min at room temperature of 20–24°C and the nailbed area was cleaned with an alcohol wipe. Drops of Optilia immersion oil fluid were then applied to each fingernail and the video capillaroscope was placed into direct contact with the nailbed. As in previous studies, the center of the nailbed and consecutive 1mm^2^ fields of the second to fifth digits were photographed.^10^ A minimum of three images were taken for each digit imaged. Nailfold video capillaroscopy was conducted by an operator who had been trained in its appropriate use.

### 2.3 Diagnoses

Five diagnoses were specified before any data collection and included diabetes, hypertension, cardiovascular events, albuminuria, and retinopathy. Diagnoses of diabetes and hypertension were based on review of each patient's chart and/or self‐report. Cardiovascular events were defined as a history of a previous myocardial infarction, stroke, unstable angina hospitalization, coronary artery disease, coronary revascularization, heart failure hospitalization, or resuscitated cardiac arrest. Albuminuria was defined as a urine albumin‐to‐creatinine ratio ≥3 mg/mmol, and retinopathy was defined as a history of photocoagulation, vitrectomy, or intravitreal injection therapy with a vascular endothelial growth factor inhibitor.

### 2.4 Machine‐learning approach

Machine‐learning models were written in PyTorch^11^ from TorchVision^12^ using Python 3.6.9. The estimation and evaluation procedures were written using the PyTorch Lightning package with evaluation metrics from Scikit‐learn.^13^ All code developed for this project is available for public review (https://github.com/hamilton-health-sciences/nailfold).

In preparation for the machine‐learning analysis, each nailfold image was labeled with the participant's ID number, sex, age, ethnicity, diabetes status (as either type 1, type 2, or no diabetes), HbA1c (as a percentage), albumin‐to‐creatinine ratio (as mg/mmol) and presence of retinopathy, hypertension, and/or cardiovascular disease status (yes/no). Albuminuria (yes/no) was defined as an albumin‐to‐creatinine ratio >3 mg/mmol, and a high HbA1c was defined as an HbA1c level ≥7.5%.

The machine‐learning analysis was carried out with convolutional neural networks,^14^ a classification model that uses images as inputs, transforms them according to nonlinear mathematical functions, and returns a prediction for each image. Patient‐level predictions were then generated by pooling predictions for all of the images for each patient.

Cross‐validation was used to assess the generalizability of the predictive model. It was applied to each of the five diagnoses (diabetes, high HbA1c, cardiovascular event, retinopathy, albuminuria, and hypertension), by splitting the patients into five randomly selected nonoverlapping subsets, with each subset containing all the capillaroscope images for the included patients. The images of four of these subsets were used to train the algorithm on the diagnosis, and the remaining images were used to test the algorithm and assess performance. This was done five times for each of the five diagnoses. Thus, all images from each patient were used to develop and assess the model. For example, if the subsets for the diabetes training were labeled A through E, subsets A, B, C, and D would have been used to generate a model that was then tested on subset E, and then subsets A, B, C, and E were used to generate a model which was then tested on subset D, and so on. Thus, for each diagnosis, the model would have been tested five times, and each time it was tested, the four subsets used for model generation were considered the training set and the fifth subset was considered the evaluation set. Each of the five models that were developed for each diagnosis was then used to classify each capillaroscope image in the evaluation set as being either positive or negative for that diagnosis. Models for hypertension, cardiovascular event status, albuminuria, and retinopathy used diabetes status as a predictor in addition to the image.

The training sets were used to select the hyperparameters for the model. Hyperparameters are the components of a machine‐learning model that cannot be learned directly from the data, such as the number of hidden layers and number of neurons per layer in a neural network. Hyperparameter optimization is an automated technique that selects the combination of hyperparameters that yields the best performance. It was achieved through random search guided by the tree‐structured Parzen estimator algorithm^15^ and implemented in Ray Tune.^16^ Neural network architectures were selected from the ResNet family of computer vision models (ResNet‐50, ResNet‐101, ResNeXt‐50, ResNeXt‐101), with architecture choice viewed as an independent hyperparameter during hyperparameter optimization. Lastly, image augmentation for these analyses was done using Kornia.^17^ This involved creating slightly modified copies of the existing images by randomly rotating or reflecting the image seen by the system during training to ensure that the model was independent of the image orientation.

### 2.5 Statistical analysis

Categorical variables were summarized using the number and percentage in each group and continuous variables were summarized by calculating each variable's mean and standard deviation.

The performance of each model for a particular diagnosis was assessed by estimating the area under the receiver operating characteristics curves (AUROC) for each of the five models (and calculating their arithmetic mean), and the area under the precision recall curves (AUPR) for each of the five models (and calculating their arithmetic means). A total of 95% confidence intervals (CIs) for these two statistics for each model were generated after reestimating them 1000 times using bootstrapping with replacement. The mean ROC curves for each diagnosis were derived by combining the five ROC curves from the five test sets as previously described.^18^ The optimal sensitivity and specificity of each model for its respective diagnosis was estimated from the mean ROC curve using Youden's index.^19^

## 3 RESULTS

A total of 5326 nailfold images were acquired from 120 participants (69.2% male) whose mean age (SD) was 60.5 (10.6) years (Table 1). A total of 60 (50%) participants had diabetes, which was by design split equally into type 1 (*N* = 30) and type 2 (*N* = 30) diabetes. Of the 107 participants with an available HbA1c level, 35.5% had a value ≥7.5% and 57.9% had a value between 6.5% and 7.5%. A total of 60 (50%) of all participants reported a previous cardiovascular event, 71 (59.2%) had previous hypertension, 32 (26.7%) had evidence of albuminuria, and 29 (24.2%) reported previous retinopathy. A mean of 44.4 images per participant were available for analysis.

**TABLE 1:** Patient characteristics

Baseline patient characteristics of all participants and number of images obtained per diagnosis	Patients *N* = 120	Images/each diagnosis *N* = 5326
Mean age (SD)	60.5 (10.6)	N/A
Males	83 (69.2)	N/A
White	111 (92.5)	N/A
Statin therapy	84 (70)	N/A
Angiotensin‐converting enzyme inhibitor/angiotensin receptor blocker therapy	72 (60)	N/A
Diabetes history	60 (50.0)	2619
Type 1 diabetes	30 (25.0)	1352
Type 2 diabetes	30 (25.0)	1267
Diabetes duration in years (SD)	27.4 (12.7)	N/A
HbA1c ≥ 7.5%^a^	38 (35.5)	1649
HbA1c ≥ 6.5%^a^	62 (57.9)	2699
Cardiovascular event	60 (50.0)	2605
Hypertension	71 (59.2)	2970
Creatinine	94 (36.0)	N/A
Albuminuria (urine albumin/creatinine >3 mg/mmol)^b^	32 (26.7)	1385
Retinopathy	29 (24.2)	1356

^a^ Only 107 patients had an HbA1c available: the 60 with diabetes and 47 without diabetes.

^b^ Only 6 people without diabetes had albuminuria.

Abbreviation: HbA1C, glycosylated hemoglobin.

**FIGURE 1:**
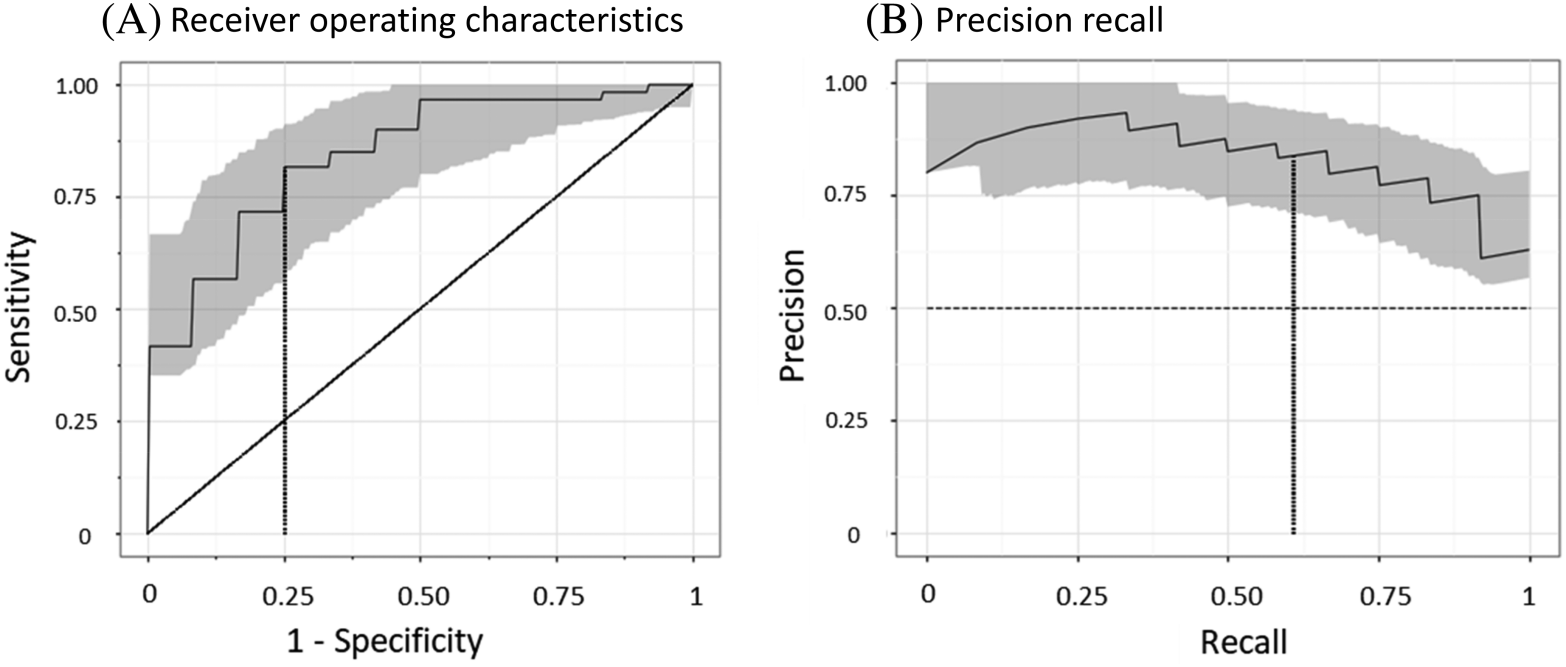
An example of a nailfold photograph from a patient without diabetes and a patient with diabetes is provided. HbA1C, glycosylated hemoglobin

**FIGURE 2:**
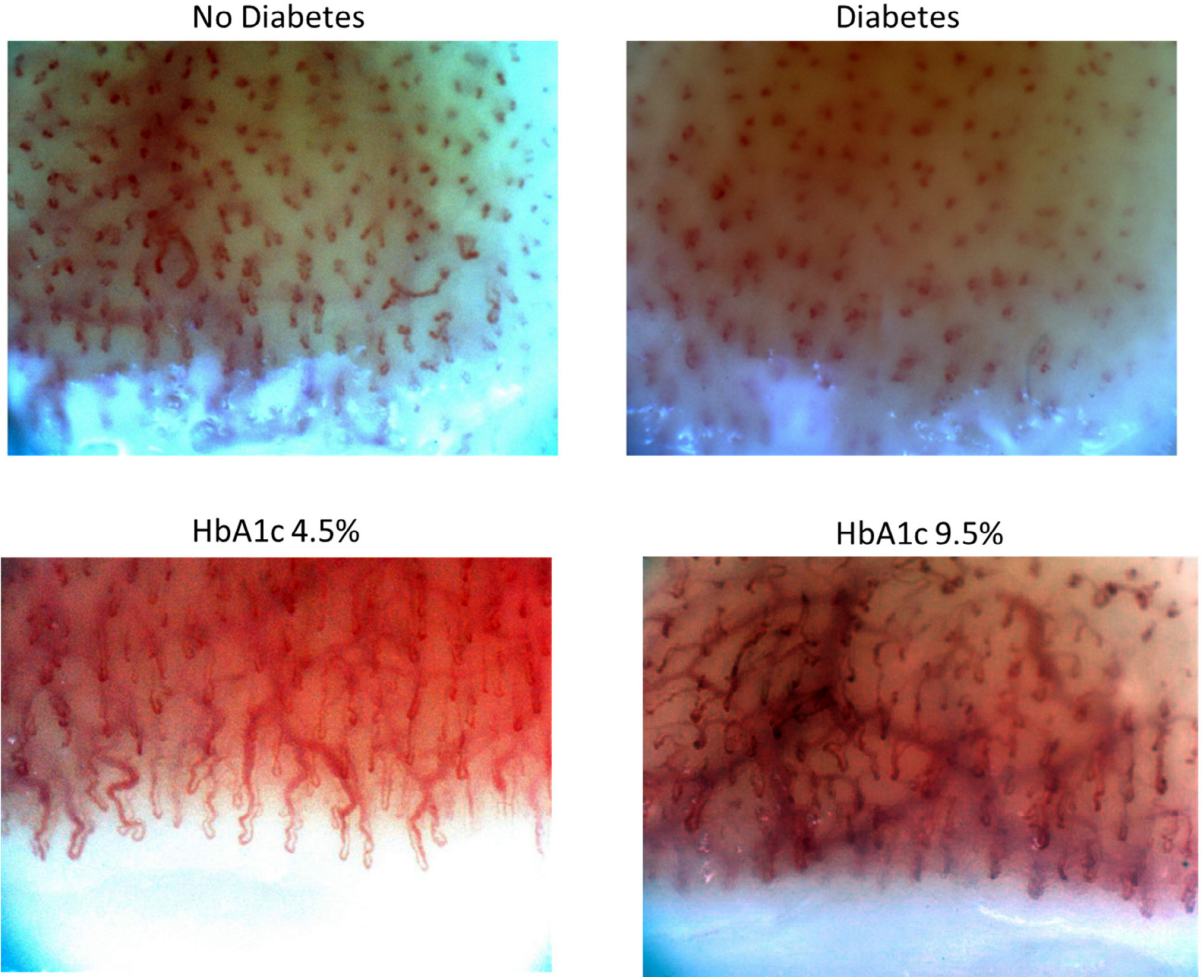
The receiver operating characteristics (Panel A) and precision recall (Panel B) curves for diabetes versus no diabetes are shown with the shaded areas denoting 95% confidence intervals. The vertical line indicates the point with the highest sensitivity and specificity

The ability of the machine‐learning models to discriminate between people with and without diabetes, a high HbA1c, hypertension, a history of a cardiovascular event, albuminuria, and retinopathy is shown in Table 2. The models were best able to identify the presence of any diabetes (ie, either type 1 or 2), yielding AUROC and AUPR curves of 0.84 (95% CI 0.76, 0.91) and 0.84 (95% CI 0.78, 0.93) respectively (Figure 1). The sensitivity and specificity of the model for diabetes was 0.82 and 0.75 respectively. Similar performance was achieved for an HbA1c ≥6.5% and for type 1 (AUROC 0.81 and AUPR 0.72) and type 2 diabetes (AUROC 0.88 and AUPR 0.81). Figure 2 shows an example of the nailfold image for a selected individual with diabetes and another one without diabetes.

**TABLE 2:** Performance metrics for diagnoses based on all images for each patient

Patients	Diagnosis	AUROC (95% CI)	AUPR (95% CI)	Sens	Spec
All patients	Diabetes	0.84 (0.76, 0.91)	0.84 (0.78, 0.93)	0.82	0.75
	HbA1c ≥7.5%	0.68 (0.57, 0.78)	0.56 (0.47, 0.74)	0.74	0.61
	HbA1c ≥6.5%	0.77 (0.66, 0.87)	0.83 (0.74, 0.91)	0.69	0.78
	Hypertension	0.51 (0.40, 0.62)	0.62 (0.54, 0.75)	0.67	0.50
	CV event	0.57 (0.47, 0.67)	0.60 (0.53, 0.73)	0.51	0.66
	Albuminuria	0.65 (0.54, 0.76)	0.44 (0.35, 0.61)	0.81	0.55
Diabetes	Hypertension	0.38 (0.19, 0.57)	0.68 (0.59, 0.84)	0.12	1.0
	CV event	0.65 (0.51, 0.78)	0.72 (0.62, 0.88)	0.52	0.86
	Albuminuria	0.45 (0.30, 0.61)	0.47 (0.38, 0.65)	0.75	0.33
	Retinopathy	0.60 (0.43, 0.76)	0.63 (0.49, 0.82)	0.57	0.75
No diabetes	Hypertension	0.50 (0.33, 0.66)	0.60 (0.48, 0.78)	0.22	1.0
	CV event	0.51 (0.36, 0.65)	0.62 (0.51, 0.77)	0.51	0.75

Abbreviations: AUPR, area under the precision recall; AUROC, area under the receiver operating characteristics; CI, confidence interval; CV, cardiovascular; HbA1C, glycosylated hemoglobin.

The machine‐learning models could not effectively identify hypertension and had only modest ability to identify people with a previous cardiovascular event, retinopathy, or albuminuria (Table 2). Notably, the model's ability to identify people with a previous cardiovascular event in the subset of individuals with diabetes was better than it was for all participants, with AUROC and AUPR curves of 0.65 (95% CI 0.51, 0.78) and 0.72 (95% CI 0.62, 0.88) respectively.

## 4 DISCUSSION

The relationship between capillary disease and long‐term diabetes consequences suggests that capillary morphology may contain diagnostic or prognostic information. This cross‐sectional analysis of nailfold capillaroscope images shows that a deep learning approach can be used to distinguish between people with and without diabetes. It also demonstrates that it can identify people whose HbA1c level is in the diabetes range and suggests that in the subset of people with diabetes, it may identify individuals with cardiovascular disease. The observation that dysglycemia is reflected in morphologic abnormalities, easily visualized in nailfold capillaries, suggests that machine‐read images of these capillary beds may have both diagnostic and prognostic value.

The harmful effect of hyperglycemia on small vessels has been recognized since the development of the ophthalmoscope in the middle of the 19th century^20^ and the recognition of arteriolar and capillary pathology in the retinae of people with hyperglycemia. Indeed, the glycemic thresholds that are currently used to diagnose diabetes are based on glucose levels that are associated with a high prevalence of these retinal vascular abnormalities.^21^ Moreover, much evidence shows that the capillary disease apparent in the retina is distributed throughout the body, including the brain, kidney, heart, and elsewhere,^5^ and is linked to strokes,^22^ cognitive decline,^22^ cardiovascular disease,^23^, ^24^ kidney disease, and other long‐term consequences of diabetes.

Nailfold video capillaroscopy has been used extensively by rheumatologists in the assessment of connective tissue diseases, with nailfold changes being associated both with disease presence and disease severity.^25^ Several studies have attempted to examine nailfold video capillaroscopic changes in people with diabetes^26^, ^27^, ^28^, ^29^, ^30^, ^31^, ^32^ and associations with complications such as retinopathy. Previous methods have relied on manual image analysis and qualitative or semiquantitative assessment of nailfold changes (such as capillary density, distribution, and morphology) that are difficult to standardize. Moreover, the literature has yielded mixed results about the association between nailfold changes and diabetes complication,^26^, ^27^, ^28^, ^29^, ^30^, ^31^, ^32^ perhaps related to variability in approaches to image acquisition and analysis. Our deep learning approach offers promise as a more scalable and generalizable method that is not reliant on manual scoring.

The link between diabetes and retinal changes, and the widespread availability of retinal images, has prompted many studies exploring the value of machine learning based on these images. For example, in one study the application of machine learning to 115 344 retinal images from 57 672 patients discriminated diabetes from nondiabetes with an AUROC of 0.85. It also predicted a 2‐fold higher incident of future diabetes.^33^ Other studies have reported that machine‐learning models can predict incidence cardiovascular events from retinal photographs.^34^

The absence of large image repositories has precluded similar machine‐learning analyses of nailfold capillary images. However, these findings add to prior manual assessments of nailfold images^30^, ^35^ and support ongoing studies of the role of machine learning for reading and interpreting such images. The fact that nailfold capillaries can be imaged easily, without the need for intravitreal dye injection and high‐cost equipment needed to properly image retinal capillaries, further supports such studies and suggests that diagnostic and prognostic information in nailfold capillary images may supplement the information that is being read in retinal images.

Our proof‐of‐concept findings are limited by the small sample size, the highly selected population, the preponderance of males and White participants, and the absence of images from other organs beds such as the retina. They are also limited by the combination of different cardiovascular outcomes into one category of a cardiovascular event. These limitations may dilute the findings, leading to underestimates of the strength of the relationship between the images and specific cardiovascular outcomes. Similarly, the cardiovascular disease and retinopathy outcomes were established by history and not by active measurement, which may have also resulted in a more conservative estimate than the actual association. Nevertheless, our results clearly demonstrate the potential of deep learning for identifying people with tissue damage from diabetes based on nailfold images and for possibly identifying those most likely to have diabetes‐related cardiovascular and other chronic consequences. They also clearly support the need to collect additional, more granular data to better elucidate the potential diagnostic and prognostic value of nailfold images.

## AUTHOR CONTRIBUTIONS

Reema Shah and Hertzel C. Gerstein designed the study, Reema Shah collected the data, and Walter Nelson, Jeremy Petch, and Karsten Roth did the machine‐learning analyses. Hertzel C. Gerstein, Jeremy Petch, and Walter Nelson wrote the first draft of the paper, and Reema Shah, Karsten Roth, Michael D. Noseworthy, and Marzyeh Ghassemi critically revised the paper. Hertzel C. Gerstein is the guarantor of the study and made the final decision to submit and publish the manuscript.

## FUNDING INFORMATION

No grants or fellowships were provided to support the writing of this paper.

## CONFLICT OF INTEREST

Jeremy Petch reports research grants from Roche and Pentavere Research Group, and in‐kind contributions to research from CloudDx. Hertzel C. Gerstein holds the McMaster‐Sanofi Population Health Institute Chair in Diabetes Research and Care. He reports research grants from Eli Lilly, AstraZeneca, Merck, Novo Nordisk, and Sanofi; honoraria for speaking from Boehringer Ingelheim, Eli Lilly, Novo Nordisk, Sanofi, DKSH, Roche, and Zuellig; and consulting fees from Abbott, Covance, Eli Lilly, Novo Nordisk, Sanofi, Pfizer, Viatris, Kowa, and Hanmi. Michael D. Noseworthy is the CEO and cofounder of TBIfinder, Inc. (https://www.tbifinder.com), a data analytics company. Reema Shah, Walter Nelson, Karsten Roth, and Marzyeh Ghassemi have nothing to disclose.
